# Supplementary material for: Time‑resolved multi-omic analysis of paclitaxel exposure in human iPSC‑derived sensory neurons unveils mechanisms of chemotherapy‑induced peripheral neuropathy
Source: Cell Death Dis. 2026 Feb 10;17(1):211. doi: 10.1038/s41419-026-08445-2 (PMC12921266; doi:10.1038/s41419-026-08445-2)
Supplement: Supplementary file 15 — Dataset 1. [file 41419_2026_8445_MOESM15_ESM.zip › CDD_Open_Data/04_RNA_Seq/Timeline_panel_plots_etc_2023-01-23_1609.zip!/Panel_Plots_etc/Evidence_Plots/post5d/evidence_plot_standard contrasts_msigdb_go_bp_5d_PTX_vs_DMSO_ID6_M12546_GOBP_ER_NUCLEUS_SIGNALING_PATHWAY.pdf]

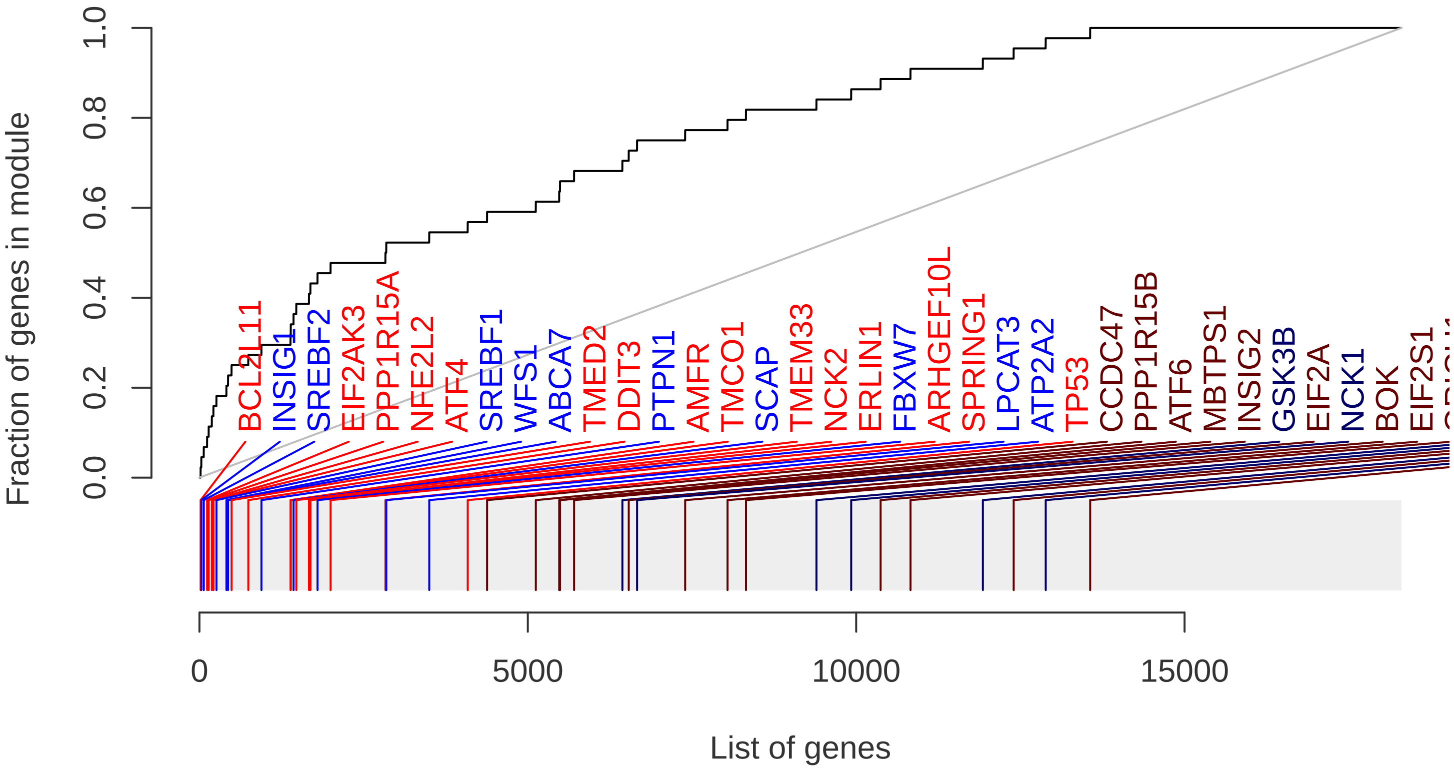

Fraction of genes in module
0.0
0.2
0.4
0.6
0.8
1.0
BCL2L11
INSIG1
SREBF2
EIF2AK3
PPP1R15A
NFE2L2
ATF4
SREBF1
WFS1
ABCA7
TMED2
DDIT3
PTPN1
AMFR
TMCO1
SCAP
TMEM33
NCK2
ERLIN1
FBXW7
ARHGEF10L
SPRING1
LPCAT3
ATP2A2
TP53
CCDC47
PPP1R15B
ATF6
MBTPS1
INSIG2
GSK3B
EIF2A
NCK1
BOK
EIF2S1
0
5000
10000
15000
List of genes
